# Loss of periactive-zone scaffold proteins does not enhance neurotransmission defects in *cla-1(ΔL)* mutants

**DOI:** 10.17912/micropub.biology.002331

**Published:** 2026-10-05

**Authors:** Elaina Cote, Zhao Xuan

**Affiliations:** 1 School of Biology and Ecology, University of Maine, Orono, ME, United States

## Abstract

Efficient synaptic transmission requires coordinated exocytosis and endocytosis at the active zone and adjacent periactive zone, but whether scaffold proteins from these two presynaptic compartments function cooperatively remains unclear. Because the active-zone scaffold CLA-1 L genetically interacts with the periactive-zone scaffolds EHS-1 and ITSN-1 during presynaptic ATG-9 sorting, we tested whether these interactions extend to neurotransmission using aldicarb assays. Although *cla-1 (ΔL)* , *ehs-1 (null)* , and *itsn-1 (null)* mutants each exhibited mild aldicarb resistance, neither *ehs-1 (null)* nor *itsn-1 (null)* enhanced the neurotransmission defect of *cla-1 (ΔL)* mutants.

**Figure 1. Loss of ehs-1 or itsn-1 does not enhance the neurotransmission defect of c la-1(ok560) mutants:** A-B. Fraction of moving animals from each genotype on 0.625 mM aldicarb at the indicated time points. Data are from four independent blinded experiments with ~30 animals per genotype per experiment. Colored asterisks indicate comparisons with N2 : *P<0.05, **P<0.01, determined by two-way repeated-measures ANOVA followed by Dunnett's multiple-comparisons test. Error bars represent SEM. C. Fraction of moving animals from each genotype on 0.05 mM levamisole at the indicated time points. Data are from three independent blinded experiments with 15 animals per genotype per experiment. No significant differences were detected between N2 and any mutant genotype (two-way repeated-measures ANOVA followed by Dunnett's multiple-comparisons test). Error bars represent SEM.

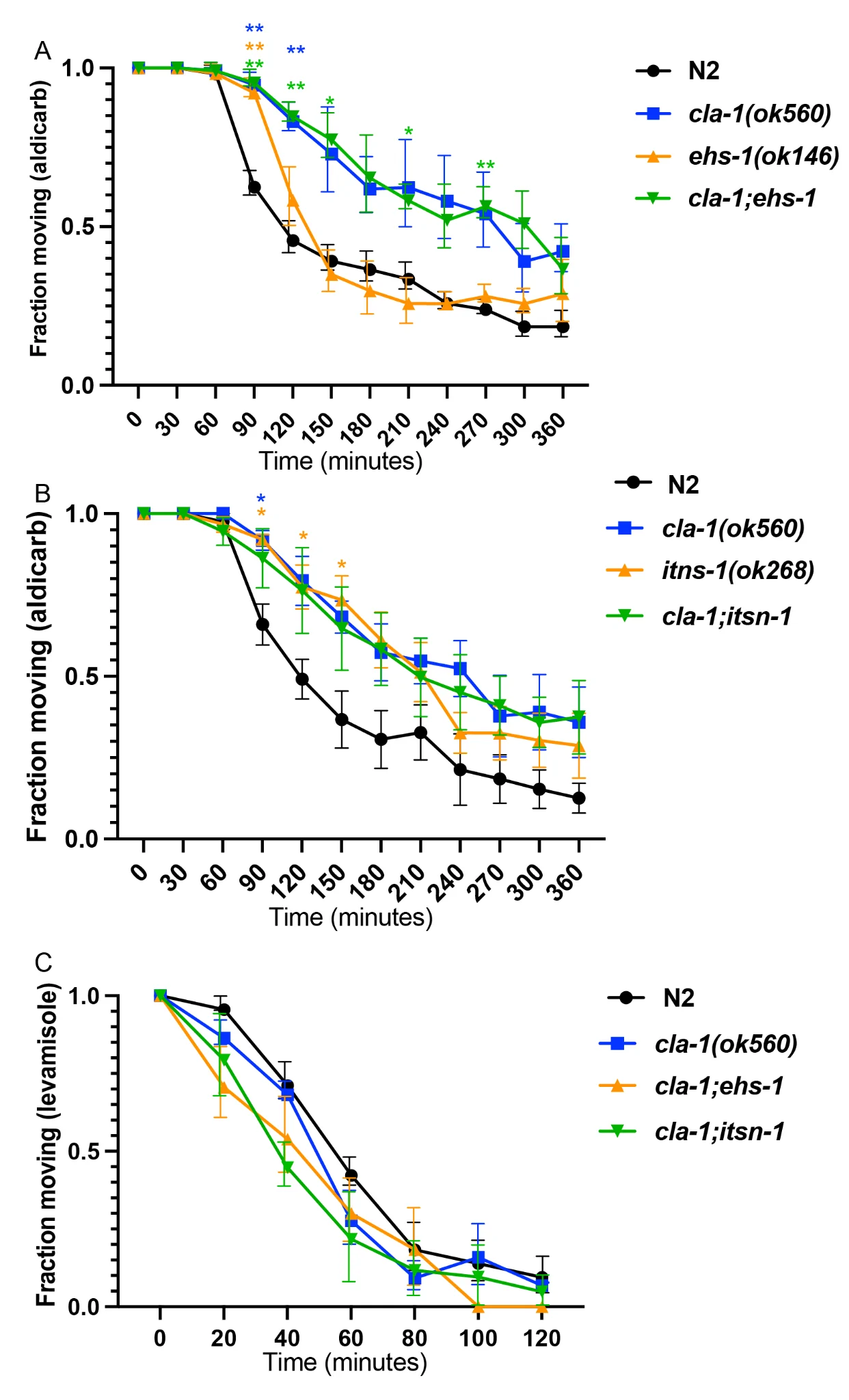



## Description

At the presynaptic site, neurotransmitters are released via synaptic vesicle exocytosis at the active zone, followed by vesicle retrieval through endocytosis at the adjacent periactive zone to sustain neurotransmission (Del Signore et al., 2023; Gad et al., 1998; Maritzen & Haucke, 2018; Roos & Kelly, 1999). Efficient synaptic transmission therefore requires coordinated organization of these two presynaptic compartments (Cano & Tabares, 2016). While genetic interactions among proteins within the active-zone exocytic machinery or the periactive-zone endocytic machinery have been examined (Maruyama et al., 2001; Schuske et al., 2003), whether scaffold proteins function cooperatively across these two presynaptic compartments remains largely unknown.

The long isoform of Clarinet ( CLA-1 L) is a large active zone scaffold in *C. elegans* that promotes synaptic vesicle clustering (Xuan et al., 2017). Eps15 ( EHS-1 in *C. elegans* ) and intersectin ( ITSN-1 in *C. elegans* ; Dap160 in *Drosophila* ) are multidomain periactive-zone scaffolds that coordinate endocytic machinery to ensure efficient cargo internalization, with conserved roles in vesicle recycling and synapse development (Koh et al., 2007; Pechstein et al., 2010; Salcini et al., 2001). Previous studies showed that CLA-1 L is required for normal presynaptic accumulation of ITSN-1 (Krout et al., 2026) and genetically interacts with EHS-1 and ITSN-1 during presynaptic sorting of the transmembrane protein ATG-9 (Xuan et al., 2023). These findings raised the possibility that active-zone and periactive-zone scaffolds cooperate in multiple presynaptic processes. We therefore asked whether the previously identified interactions also extend to neurotransmission by testing the effects of simultaneously disrupting CLA-1 L and either EHS-1 or ITSN-1 .

The aldicarb assay is a widely used behavioral assay in *C. elegans* to assess synaptic transmission at the neuromuscular junction (Bera et al., 2025; Hu et al., 2011; Mahoney et al., 2006). The assay measures the sensitivity of worms to the paralyzing effects of the acetylcholinesterase inhibitor, aldicarb. Mutations that reduce presynaptic acetylcholine release confer resistance to aldicarb (Nonet et al., 1998), whereas mutations that enhance acetylcholine release increase aldicarb sensitivity (McEwen et al., 2006). Although aldicarb sensitivity can also be influenced by inhibitory GABAergic transmission (Locke et al., 2008), the assay provides a functional readout of neurotransmission at the neuromuscular junction. We examined *cla-1 ( ok560 )* , which disrupts the long isoform of CLA-1 (Xuan et al., 2017), the null alleles *ehs-1 ( ok146 )* (Salcini et al., 2001) and *itsn-1 ( ok268 )* (Rose et al., 2007), and the double mutants. While *cla-1 ( ok560 )* and *itsn-1 ( ok268 )* exhibited increased aldicarb resistance relative to N2 , ehs-1 *( ok146 )* showed resistance, only at the 90-min time point ( Figure 1A and B). Importantly, neither the *cla-1 ( ok560 ); ehs-1 ( ok146 )* nor *cla-1 ( ok560 ); itsn-1 ( ok268 )* double mutant showed enhanced aldicarb resistance relative to *cla-1 ( ok560 )* at any time point ( Figure 1A and B). Furthermore, the aldicarb resistance observed in *cla-1 ( ok560 )* and the double mutants was not accompanied by detectable levamisole resistance ( Figure 1C ), indicating that the observed phenotypes are unlikely to result from impaired postsynaptic nicotinic acetylcholine receptor function under the conditions tested (Fleming et al., 1997; Gally et al., 2004).

Despite synergistic effects on ATG-9 mislocalization (Xuan et al., 2023), loss of *ehs-1* or *itsn-1* did not significantly enhance the aldicarb resistance of *cla-1 ( ok560 )* mutants. These findings suggest that CLA-1 L and these periactive-zone scaffolds might function in the same process or pathway supporting neurotransmission. The contrast between the synergistic effects on ATG-9 sorting and the lack of enhancement in aldicarb sensitivity suggests that genetic interactions between these scaffolds may depend on the presynaptic process or functional output being examined. Although the aldicarb assay does not directly measure exo-endocytosis coupling, it provides an initial functional test of whether interactions between active-zone and periactive-zone scaffold proteins influence the behavioral output of synaptic transmission. Future studies testing genetic interactions between periactive-zone scaffold proteins and other active-zone scaffold proteins that are not implicated in endocytic function, such as ELKS-1 (homologous to vertebrate ELKS/CAST/ ERC2 and *Drosophila* Bruchpilot) (Deken et al., 2005; Held & Kaeser, 2018), will help determine whether the lack of genetic enhancement is unique to CLA-1 L or represents a more general property of active-zone scaffolds.

Previous studies reached different conclusions regarding the role of ITSN-1 in neurotransmission, reporting either aldicarb hypersensitivity (Rose et al., 2007) or reduced spontaneous synaptic transmission (Wang et al., 2008). In the latter study, electrophysiological and ultrastructural analyses suggested that ITSN-1 and EHS-1 function similarly during synaptic vesicle endocytosis. Our finding that *itsn-1 ( ok268 )* exhibits aldicarb resistance differs from the aldicarb hypersensitivity reported by Rose et al. (2007), but is consistent with the reduced synaptic transmission observed by Wang et al. (2008). The reason for the discrepancy between our aldicarb results and those of Rose et al. (2007) remains unclear and could reflect differences in genetic background or experimental conditions. A limitation of this study is that only a single allele of each gene was examined; therefore, contributions from unrelated background mutations cannot be excluded. Analysis of additional independent alleles will be important to determine whether the observed phenotypes are specifically attributable to loss of the corresponding genes.

## Methods

*C. elegans* strains : Worms were raised on NGM plates at 20 °C using OP50 Escherichia coli as a food source. N2 Bristol was used as the wild-type reference strain. *cla-1 ( ok560 )* was outcrossed ten times to the N2 Bristol background, *ehs-1 ( ok146 )* was outcrossed twice, and *itsn-1 ( ok268 )* was not outcrossed prior to use. Double-mutant strains were generated by genetic crosses between the corresponding single-mutant strains.

**Table:** 

**Strain**	**Genotype**	**SOURCE**
**DCR4957**	*cla-1 ( ok560 ) IV *	Colón-Ramos lab
**NM1568**	*ehs-1 ( ok146 ) II *	CGC
**VC201**	*itsn-1 ( ok268 ) IV *	CGC
**ZJX16**	*cla-1 ( ok560 ); ehs-1 ( ok146 ) *	Xuan lab
**ZJX11**	*cla-1 ( ok560 ); itsn-1 ( ok268 ) *	Xuan lab

Genetic validation of mutant strains:

All mutant alleles examined in this study are deletion alleles. Deletion mutations were validated by PCR using a three-primer genotyping strategy. For each allele, a common forward primer was paired with either a WT-detection reverse primer or a mutation-detection reverse primer to distinguish the wild-type and deletion alleles. Double-mutant strains generated by genetic crosses were confirmed by independently genotyping each mutant allele. Genomic sequences used for primer design were obtained from WormBase (release WS298 ), and genotyping primers were designed using ApE (A Plasmid Editor, version2.0.50b3). Primer sequences used for genetic validation are provided below.

**Table:** 

**Gene (allele)**	**Common forward (5′–3′)**		**WT-detection reverse (5′–3′)**	**Mutation-detection reverse (5′–3′)**
***cla-1 ( ok560 ) ***		tctgctcttcctccaacacc	ttgcatccgctatttcttc	gtcagctcccgattgcac
***ehs-1 ( ok146 ) ***		aacaatcttctccagctcatcc	tttccgctccaccagcattg	ctcagttggttcctgacgtg
***itsn-1 ( ok268 ) ***		caacgtcagcaacgagaaaa	gctcatcctcggatcttgcttc	tcgggaaccatccaatttcg

Aldicarb assays : Animals were assayed for acute exposure to aldicarb (Mahoney et al., 2006). Aldicarb (Sigma-Aldrich) was prepared as a stock solution of 200 mM stock in 50% ethanol. Aldicarb sensitivity was measured by transferring 30 animals to 60mm plates containing 0.625 mM aldicarb (final ethanol concentration, 0.16% [v/v]) and then assaying the time course of paralysis. Animals were considered paralyzed once they no longer moved even when prodded with a platinum wire three times on the head and tail. The fraction of animals moving to the total number of animals on the plate was calculated for each time point. L4 worms were picked the day before the assay. All assays were performed blinded to genotype.

Levamisole assay :

Animals were assayed for defects in postsynaptic nicotinic acetylcholine function by exposure to the nACh receptor agonist, levamisole. Levamisole (VWR) was prepared in M9 buffer at a concentration of 0.05 mM and distributed into 12-well plates. Five young adult animals were transferred to each well, with three wells per genotype (15 animals total per genotype per experiment), and allowed to thrash in 1 mL of 0.05 mM levamisole for 2 hours. Worms were assayed for thrashing every 20 minutes, recording the fraction of worms moving at each time point. Young adult worms were obtained by picking L4 animals the day prior to experimentation. Assays were performed blinded to genotype.

Statistical analysis : Data are presented as mean ± SEM from four independent blinded experiments for each aldicarb assay ( Figure 1A, B) and three independent blinded experiments for the levamisole assay ( Figure 1C ). For comparisons between each mutant genotype and N2 , aldicarb and levamisole time-course data were analyzed by two-way repeated-measures ANOVA followed by Dunnett's multiple-comparisons test, with N2 designated as the control. For the aldicarb assays, to test whether loss of *ehs-1* or *itsn-1* enhanced the *cla-1 ( ok560 )* phenotype, *cla-1 ( ok560 )* was compared with each corresponding double mutant in separate two-way repeated-measures ANOVAs followed by Šídák's multiple-comparisons test. Statistical analyses were performed using GraphPad Prism version 11.0.2 (GraphPad Software, Boston, MA, USA).
